# Supplementary material for: Prevalence and determinants of low testosterone levels in men with type 2 diabetes mellitus; a case-control study in a district hospital in Ghana
Source: PLOS Glob Public Health. 2021 Dec 2;1(12):e0000052. doi: 10.1371/journal.pgph.0000052 (PMC10021198; doi:10.1371/journal.pgph.0000052)
Supplement: Attachment — Submitted filename: Response to Reviewers.docx [file pgph.0000052.s003.docx]

**RESPONSES TO REVIEWERS’ COMMENTS**

Manuscript title:

Manuscript ID: PGPH-D-21-00300

Dear Editors and Reviewers,

We greatly appreciate the thorough and thoughtful comments provided on our submitted manuscript. We have addressed and revised accordingly all the comments of the reviewer(s).

Attached below are the detailed responses to the entire reviewer’s comments and they are tracked in the main manuscript.

Sincerely,

The Authors of the Manuscript: PGPH-D-21-00300

Reviewer #1

Type II diabetes need to be replaced with type 2 diabetes mellitus.

**Authors comment**

Type II diabetes is replaced with type 2 diabetes mellitus.

Reviewer #1

Articles needs to be written well like, line 25 The should be capital

**Authors comment**

Line 27: The sentence is capitalized

Reviewer #1

Line 374 it is written that cross sectional study and before it is written as case control study. Need a clarity on this.

**Authors comment**

Line 377: The study is a case-control study and the needed correction is made

Reviewer #1

Table 3 Odd ratio is missing.

**Authors comment**

Table 3 compared difference in mean clinical and hormonal parameters and not odds ratio. In table 4, all odds ratios are reported.

Reviewer #2:

Title: The current title is worded awkwardly and should use person-first language such as “"Prevalence and determinants of low testosterone levels in men with Type II diabetes mellitus; a case-control study in a district hospital in Ghana"

**Authors comment**

Thank you for the suggestion; the authors adopted the suggested title and modification made in the running title as well

**Reviewer #2:**

Assay: The methodological description of the assay AccuDiag Testosterone Assay Kit suggests using a total testosterone measurement, but the authors later refer to free testosterone in the results section. Could the authors clarify if they measured total, free or both testosterone levels? Male hypogonadism in insulin resistance partially involves a decrease in SHBG lowering total testosterone levels, but preserving intact free testosterone levels. This distinction is important as the ELISA assay is different than the commonly accepted LC-MS assays used in other parts of the world that report both free and total testosterone levels.

**Authors comment**

Thanks for the observation, the AccuDiag Testosterone Assay Kit measured total testosterone and it was clearly stated in line 186-188 that classification of hypogonadism was based on total testosterone. Therefore, the necessary changes have been made at the results section (line 235 and line 242).

Reviewer #2:

*Results – One of the more interesting findings of the study is that the diabetes cohort had a lower BMI than the control cohort. Could the authors comment on reasons as to why this might be a difference? Could it be attributed to stage of type 2 diabetes, poor control and subsequent glucosuria, or other factors?

**Authors comment**

The findings of the study depicted that the T2DM cohort had a lower BMI than the control cohort. Even though the average BMI of the diabetic men was significantly lower than the healthy men, both were within the normal range. This contradicts the results of [17] that reported that males with diabetes have a higher average BMI than their non-diabetic counterparts. In the pathogenesis of T2DM, lower body mass index (BMI) is consistently associated with reduced type 2 diabetes risk, among people with varied family history, genetic risk factors and weight, while in established T2DM patients weight loss has been shown to meliorate glycaemic control, with severe calorie restraint even reversing the progression of T2DM [23,24]. The mechanisms for this BMI paradox are not fully understood, but proposed explanations include T2DM individuals lose weight and become frail as a result of underlying illnesses that cause wasting. A study by Peyrot et al., [25] into possible psychological barriers to diabetes care, also found that many participants with T2DM felt very anxious and ashamed about their weight and thus reducing their weight reduces their experience of weight stigma.

Another possible explanation is the genetic predisposition in T2DM patients. According to Habbu et al., [26], more South Asians developed T2DM at BMI below 30 kg/m^2^ (38%) than White Europeans (26%) and African-Caribbeans (29%). This suggest a possible low BMI among T2DM patients in our study subjects. Lastly, life style interventions that target diets and weight-loss have shown demonstrable benefit for reducing the risk of type II diabetes in high-risk and pre-diabetic individuals but have not been well-studied in people at lower risk of diabetes. These findings suggest that all individuals can substantially reduce their type 2 diabetes risk through weight loss, and support the broad deployment of weight loss interventions to individuals at all levels of diabetes risk as a public health measure [24]. In our present study since our diabetic cohorts visit the clinic regularly and visit the dietician, there might be measures going on to help reduce the risk of the infection, which might not be seen in the control cohort who might be moving around freely without any restrictions on diet.

Reviewer #2:

*Results – Dysglycemia occurs in a spectrum, and the authors likely observed similar gonadal deficiencies in subjects with prediabetes as part of clinical care. Could the authors comment on how the observed association would be affected by prediabetes in their population, or as a future study?

**Authors comment**

Thank you for the comment, however, the current study participant were type 2 diabetic men and excluded pre diabetic individuals. We have therefore stated in our recommendation that the researchers will conduct further studies in the future to determine if pre diabetics aged between 20-40 years at the district hospital express similar gonadal deficiencies.

Reviewer #2:

*Reporting standards – As a reviewer I would ask the authors to complete the STROBE statement checklist for ease of review (https://www.strobe-statement.org/index.php?id=strobe-home). Most of the elements are included in the document, but the checklist would simplify review and compliance checking. Unfortunately, I was not able to review the supplemental material in the RAR or data.sav file provided.

**Authors comment**

The authors have completed the STROBE statement checklist for ease of review

Reviewer #2:

*I would suggest organizing the manuscript with the text first and tables at the end per usual manuscript submission guidelines.

**Authors comment**

Thanks for the comment, but we strictly followed the Plos guidelines, which suggest that the tables should be in-text and tables uploaded separately.

Reviewer #2:

*There are minor grammatical spelling errors that can be corrected with editorial assistance. The list below is not exhaustive:

Page 13, lines 216-217: Sentence should be corrected to “The body mass index (BMI) of the healthy controls was significantly higher than...”

**Authors comment**

Correction has been made

Reviewer #2:

Page 15, line 258: Sentence should start with “The distribution…"

**Authors comment**

Correction has been made

Reviewer #2:

Page 22: Line 326: Sentence should be corrected to “Author SD conceived the study.”

**Authors comment**

Correction has been made

**Reviewer #3:**

INTRODUCTION:

1. Line no-61: Why loss of memory, depression, lack of concentration will come first over riding the main affects of hypogonadism? I would like to request the author to organize the line accordingly with proper citation.

**Authors comment**

Line 65-67 has been modified to read as “like erectile dysfunction (ED), poor morning erection, low libido, loss of memory, physical decline, difficulty in concentration and depression”.

**Reviewer #3:**

2. What does the author mean by " Physical decline". Please clarify

**Authors comment**

Physical decline in this context means decline in strength and health in general and has been qualified appropriately in line 66.

**Reviewer #3:**

3. Type-2 DM is written differently in all over the manuscript. I would request to use one uniform pattern.

**Authors comment**

For the sake of uniformity, Type 2 diabetes mellitus has been used throughout the manuscript.

**Reviewer #3:**

STUDY PARTICIPANTS:

1. Cut off values for fasting blood sugar and HBA1c is adopted from where? Any reference, then author should mention it. Because different guidelines use different cut off values.

**Authors comment**

It was referenced under the subsection “Collection of information and patients’ selection”; however, it has also been referenced under the study participants.

**Reviewer #3:**

2. Study participant selection criteria should be more clear.

**Authors comment**

The study participant selection criteria are clarified and now read as “This study involved 150 type 2 diabetic men who had registered and receiving treatment at the diabetic clinic of Nkenkaasu Government Hospital and 150 control group comprised of apparently healthy blood donors and those visiting their relatives on admission” …….

**Reviewer #3:**

BODY MASS INDEX- Authors have categorized according to WHO BMI Category? But why no citation or acknowledgement?

**Authors comment**

Reference provided: Ephraim RKD, Awuku YA, Adu P, Ampomah LTW, Adoba P, Panford S, et al. High risk of coagulopathy among Type-2 Diabetes Mellitus clients at a municipal hospital in Ghana. Ghana Med J. 2017;51(3):101–7.

**Reviewer #3:**

BP MEASUREMENT- Line no 124 and whole procedure of BP measurement were done maintain a standard procedure. So, why no citation or acknowledgement?

**Authors comment**

Reference provided: Nkansah C, Addai-Mensah O, Mensah K, Owusu M, Ephraim RKD, Adu P, et al. Plasminogen Activator Inhibitor-1 in poorly controlled vs well controlled Type-2 Diabetes Mellitus patients: A case-control study in a district hospital in Ghana. PLoS One. 2021;16(4):e0250090

**Reviewer #3:**

BLOOD SAMPLE COLLECTION- This procedure is also done maintaining standard protocol. Is it? Clear writing and proper citation is very necessary here.

**Authors comment**

Reference provided: Nkansah C, Addai-Mensah O, Mensah K, Owusu M, Ephraim RKD, Adu P, et al. Plasminogen Activator Inhibitor-1 in poorly controlled vs well controlled Type-2 Diabetes Mellitus patients: A case-control study in a district hospital in Ghana. PLoS One. 2021;16(4):e0250090

**Reviewer #3:**

LABORATORY ASSAY- Are the whole cut off/ normal values are taken from standard guideline? Nothing is clear over there. If yes, then acknowledgement is required.

**Authors comment**

The cut off/ normal values are for Methodist Hospital Laboratory, Wenchi, Bono Region, Ghana where the analysis was done. Each laboratory validates its own reference range by collecting data from vast number of laboratory tests and the reference range is the set of values 95% of the normal population falls within.

RESULTS:

1. Most of the study participants were at the age group of 40 to 70 years. Normally gonadal function diminishes at this ages. Although a clear explanation is given on the discussion part, but i found it as not enough. It should be more clarified along with some evidences from published studies.

2. Erectile dysfunction rate is quite high and it seems normal at this age group. But the normal individual is also fall into that category. A clear explanation on this finding is very necessary to make the statement reader palatable.

**Authors comment**

The comments have been addressed at the discussion section

**Reviewer #3:**

DISCUSSION:

This part is quite good and have made many things clear. But still there are few scope to make it more synchronized with the findings from analysis.

**Reviewer #3:**

1. Most of the study participants were at the age group of 40 to 70 years. Normally gonadal function diminishes at this ages. Although a clear explanation is given on the discussion part, but I found it as not enough. It should be more clarified along with some evidences from published studies.

**Authors comment**

The study depicted that, most of our study participants had low testosterone level. Most of the study participants were at the age group of 40 to 70 years. Studies have shown that mean testicular volume and gonadal function diminishes at this ages. The mean testicular volume tends to increase between 11 and 30 years of age, remains constant between 30 and 60 years of age, and decreases gradually every year after age 60 [34]. Few data on hypogonadism in aging men are available because of the deficiency of evidence regarding the exact criteria for distinguishing testosterone deficiency in older men who do not have pathological hypogonadism [35–37]. The Massachusetts Male Aging Study, using both total testosterone and calculated free testosterone, gave crude prevalence estimates for hypogonadism in men from age 40 to 69 years, ranging at baseline from 6.0 to 12.3% [38]. This is in accordance with the results of this study. Mahmoud et al. 2003, found the mean testicular volume in men over 75 years to be 31 % less than in men between 18 and 40 years of age. This difference is associated with significantly higher mean serum levels of gonadotropins and lower serum free testosterone. Interestingly, when the incidence of hypogonadism was determined by decades, nearly all of the categories of illness were more prevalent in men aged 50–70 years [39], which is consistent with our study

**Reviewer #3:**

2. Erectile dysfunction rate is quite high and it seems normal at this age group. But the normal individual is also fall into that category. A clear explanation on this finding is very necessary to make the statement reader palatable.

**Authors comment**

Erectile dysfunction rate was quite high in our study subjects irrespective of their diabetic status and it seems normal at this age group. According to a recent analysis on the prevalence of sexual dysfunction the prevalence of ED was 1%–10% in men younger than 40 years, 2%–9% among men between 40 and 49 years, and it increased to 20%–40% among men between 60–69 years, reaching the highest rate in men older than 70 years (50%–100%) [19]. In the Massachusetts Male Aging Study, diabetic men showed a threefold probability of having ED than men without diabetes; moreover, the age-adjusted risk of ED was doubled in diabetic men compared with those without diabetes [20]. Similar erectile dysfunction rates were also found in France, where 39% of men aged 18 to 70 reported erectile dysfunction [21]. Another report by Thai Erectile Dysfunction Epidemiological Study Group (TEDES) among men aged 40 to 70 revealed an erectile dysfunction prevalence of 37.5% [22]. The findings of this study defining the age group for erectile dysfunction does not rule out ED at early or late stage, therefore categorizing our age group around 40-70 years could not change already known facts the most important factor is the stage of the diabetes. Among the diabetic patients, as age increases and/ or the condition progresses, the risk of developing peripheral neuropathy, hypertension, and impotency would also elevate, which might be the reason for an increased odds of ED.

Conclusion:

Few lines were beautifully written. But last portion is quite hazy and it demands re- write maintaining a flaws with the overall study.

**Authors comment**

Conclusion re-written to read as follows: “The study recorded a worrying prevalence of hypogonadism even among the healthy control group but with Type 2 diabetic men having a high incidence of hypogonadism. This study also demonstrates that FSH and LH concentrations are certainly lower in a relatively large number of the males with type II diabetes compared with the healthy men. Body Mass Index, smoking and leisure time activity (sedentary lifestyle) were associated with hypogonadism in the study population. Given a large number of individuals with diabetes worldwide, the high prevalence of hypogonadism in type 2 diabetes raises important issues about its possible consequences on the sexual, reproductive and general health of our study population. A further study is recommended to carry out to holistically assess the prevalence of gonadal deficiencies in subjects with prediabetes patients aged between 20-40 years at the district hospital”.

**Reviewer #3:**

ABSTRACT and TITLE: This is quite good and well Witten. But, again the conclusion should be more specified.

**Authors comment**

Title modified as “Prevalence and determinants of low testosterone levels in men with Type 2 diabetes mellitus; a case-control study in a district hospital in Ghana”

Conclusion of the abstract now read “Type 2 diabetic men have high incidence of hypogonadism, irrespective of their baseline clinical, lifestyle or demographic characteristics. Smoking and sedentary lifestyle and BMI were associated with hypogonadism in the study population. Routine testosterone assessment and replacement therapy for high risk patients is recommended to prevent the detrimental effect of hypogonadism in diabetic men”.

**Editor’s comment**

While revising your submission, please upload your figure files to the Preflight Analysis and Conversion Engine (PACE) digital diagnostic tool, https://pacev2.apexcovantage.com/. PACE helps ensure that figures meet PLOS requirements. To use PACE, you must first register as a user. Registration is free. Then, login and navigate to the UPLOAD tab, where you will find detailed instructions on how to use the tool. If you encounter any issues or have any questions when using PACE, please email PLOS at figures@plos.org. Please note that Supporting Information files do not need this step.

**Authors comment**

Figure files in pace format
