# Supplementary material for: Prevalence and determinants of low testosterone levels in men with type 2 diabetes mellitus; a case-control study in a district hospital in Ghana
Source: PLOS Glob Public Health. 2021 Dec 2;1(12):e0000052. doi: 10.1371/journal.pgph.0000052 (PMC10021198; doi:10.1371/journal.pgph.0000052)
Supplement: Attachment — Submitted filename: Response to Reviewers.docx [file pgph.0000052.s004.docx]

**RESPONSES TO REVIEWERS’ COMMENTS**

Manuscript title:

Manuscript ID: PGPH-D-21-00300

Dear Editors and Reviewers,

We greatly appreciate the thorough and thoughtful comments provided on our submitted manuscript. We have addressed and revised accordingly all the comments of the reviewer(s).

Attached below are the detailed responses to the entire reviewer’s comments and they are tracked in the main manuscript.

Sincerely,

The Authors of the Manuscript: PGPH-D-21-00300

**Reviewer #3:**

Objectives and rationale of the study is still hazy. Last part of introduction is not well written and is giving a discrete essence.

**Authors response:**

Last part of the introduction is re-written to read as “Although mediated by a variety of mechanisms, hypogonadism is more common in diabetic than in non-diabetic men in the Western world and in Asia compared to Africa This could be attributable to the paucity of data on this issue in sub-Saharan African men.

There are very little documented data in Ghana on the prevalence of hypogonadism in both Type 2 diabetic patients and healthy men, because of limited resources and cost-ineffectiveness of screening all men. To the best of our knowledge only one study is available on hypogonadism in diabetes mellitus men in Ghana [5]. This study, therefore, investigated the levels of male sex hormones and determined the prevalence of hypogonadism among Type 2 diabetic Ghanaian men in a district hospital in Ghana, and non-diabetic controls.

**Reviewer’s comment:**

#Sample size is less, so it is really difficult to address the title as prevalence study. It is only a proportion from small samples.

**Authors response**

Thank you for the comment, however the authors have their reservation on this comment. First sample size needed to draw the inference was calculated using Kelsey’s formula, based on power of 80% and α of 0.05. The prevalence of hypogonadism in Ghanaian men with diabetes is 35.2%, and in control group is 6.7% according to Asare-Anane et.al., [5]. From the formula above, the minimum number of T2DM required for this study was 33 with corresponding controls of 33. However, this study employed 300 subjects: 150 T2DM patients and 150 healthy controls. Hence making it possible for the authors to rightfully use prevalence in our context.

**Reviewer’s comment:**

#Age group of the study may create a dilemma.

**Authors response:**

We have acknowledged the “age group” in our limitation. “The study was also limited by the advanced age of the participants (41-70 years), hence the high prevalence of hypogonadism may have been masked by the age bracket of the study participants. To help address this limitation, we recommend future studies to consider participants below 40 years of age. In the face of these limitations, this study gives significant data for the occurrence and predictors of hypogonadism among Ghanaian men with T2D”.

**Reviewer’s comment:**

#Could not co-relate the line no 130 with the citation number 14.

**Authors response:**

Line 125-131 rather describes Blood Pressure measurement using Korotkoff 1 and 5 as referenced from Citation 14

**Reviewer’s comment:**

#Line 193-195 demands an acknowledgement, but this is absent there i see.

**Authors response:**

Acknowledgement is on lines 458-460
